# AHCODA-DB: a data repository with web-based mining tools for the analysis of automated high-content mouse phenomics data

**DOI:** 10.1186/s12859-017-1612-1

**Published:** 2017-04-04

**Authors:** Bastijn Koopmans, August B. Smit, Matthijs Verhage, Maarten Loos

**Affiliations:** 1 Sylics (Synaptologics BV), Amsterdam, The Netherlands; 2 Department of Molecular and Cellular Neurobiology, Center for Neurogenomics and Cognitive Research (CNCR), Amsterdam Neuroscience, VU University Amsterdam, Amsterdam, The Netherlands; 3 Department of Functional Genomics, Center for Neurogenomics and Cognitive Research (CNCR), Amsterdam Neuroscience, VU University Amsterdam, Amsterdam, The Netherlands; 4 Department of Clinical Genetics, VU Medical Center, Amsterdam, The Netherlands

**Keywords:** Data analysis, Database, Neuroscience, Statistics, Visualization, Mouse behaviour, AHCODA, Phenotyping

## Abstract

**Background:**

Systematic, standardized and in-depth phenotyping and data analyses of rodent behaviour empowers gene-function studies, drug testing and therapy design. However, no data repositories are currently available for standardized quality control, data analysis and mining at the resolution of individual mice.

**Description:**

Here, we present AHCODA-DB, a public data repository with standardized quality control and exclusion criteria aimed to enhance robustness of data, enabled with web-based mining tools for the analysis of individually and group-wise collected mouse phenotypic data. AHCODA-DB allows monitoring in vivo effects of compounds collected from conventional behavioural tests and from automated home-cage experiments assessing spontaneous behaviour, anxiety and cognition without human interference. AHCODA-DB includes such data from mutant mice (transgenics, knock-out, knock-in), (recombinant) inbred strains, and compound effects in wildtype mice and disease models. AHCODA-DB provides real time statistical analyses with single mouse resolution and versatile suite of data presentation tools. On March 9th, 2017 AHCODA-DB contained 650 k data points on 2419 parameters from 1563 mice.

**Conclusion:**

AHCODA-DB provides users with tools to systematically explore mouse behavioural data, both with positive and negative outcome, published and unpublished, across time and experiments with single mouse resolution. The standardized (automated) experimental settings and the large current dataset (1563 mice) in AHCODA-DB provide a unique framework for the interpretation of behavioural data and drug effects. The use of common ontologies allows data export to other databases such as the Mouse Phenome Database. Unbiased presentation of positive and negative data obtained under the highly standardized screening conditions increase cost efficiency of publicly funded mouse screening projects and help to reach consensus conclusions on drug responses and mouse behavioural phenotypes. The website is publicly accessible through https://public.sylics.com and can be viewed in every recent version of all commonly used browsers.

## Background

Mouse models of human brain disorders play an important role in understanding disease mechanisms and in preclinical development of therapeutic strategies. Whereas many molecular processes have been studied systematically on a large scale using –omics approaches for decades, the methodology of studying behavioural phenotypes (behavioural phenomics) has become available only recently. It is widely recognized that in-depth and well-controlled characterisation of animal behaviour is essential for comprehensive understanding of mouse phenotypes and pharmacological responses [1]. Therefore, efficient sequential batteries of behavioural tests have been used to obtain high-content phenomic profiles of mouse models and pharmacological responses. In addition, automated home-cage approaches have been developed that test many aspects of mouse behaviour in a highly standardized manner without human intervention. These automated tools for behavioural phenotyping generate hundreds of behavioural parameters [2–5], not only increasing the quantity of data obtained, but also quality, due to rigorous standardization and lack of human interference.

Despite these advances in obtaining high-content behavioural profiles, systematically mining the data for genetic effects and pharmacological responses remains a challenge, in contrast to other –omics platforms with public data repositories and user friendly tools (e.g. Gene expression omnibus, Allen Brain Atlas). Although several repositories are available to archive and mine qualitative data on mouse mutants (e.g. MGI website [6]) and precomputed group averages of inbred mouse lines (e.g. WebQTL [7], the Mouse Phenome Database [8], the International Mouse Phenotyping Consortium [9]), no repository is currently available for quantitative high-content mouse phenomics data other than the supplementary data of scientific publications. Even more important, the tools for systematic, large-scale data mining of phenomics profiles to delineate similarities and differences between novel and established mouse models and pharmacological interventions are lacking. Therefore valuable data becomes untraceable and not used by the research community. Furthermore, an increasing number of laboratories is using standardized home-cage testing protocols that produce highly standardized output. However, a platform for storage and comparison of this standardized data obtained by different laboratories is currently lacking. To offer an open access repository with web-based mining tools for the wealth of quantitative data gathered by individual laboratories and international research consortia using both automated home-cages and conventional tests and at the resolution of individual mice, we established “AHCODA-DB”. Open accessibility at the resolution of individual mice enhances transparency (i.e. enables in depth post-publication peer review to enhance reproducible science), and allows (meta) analyses to generate and test new hypothesis [10]. This resource and related tools should allow individual scientists and consortia conducting experiments with common inbred strains and/or mutant lines, with and without drug treatment to analyse and systematically compare their data across time and experiments, with reference to standard collected data.

## Construction and content

**Fig. 1:**
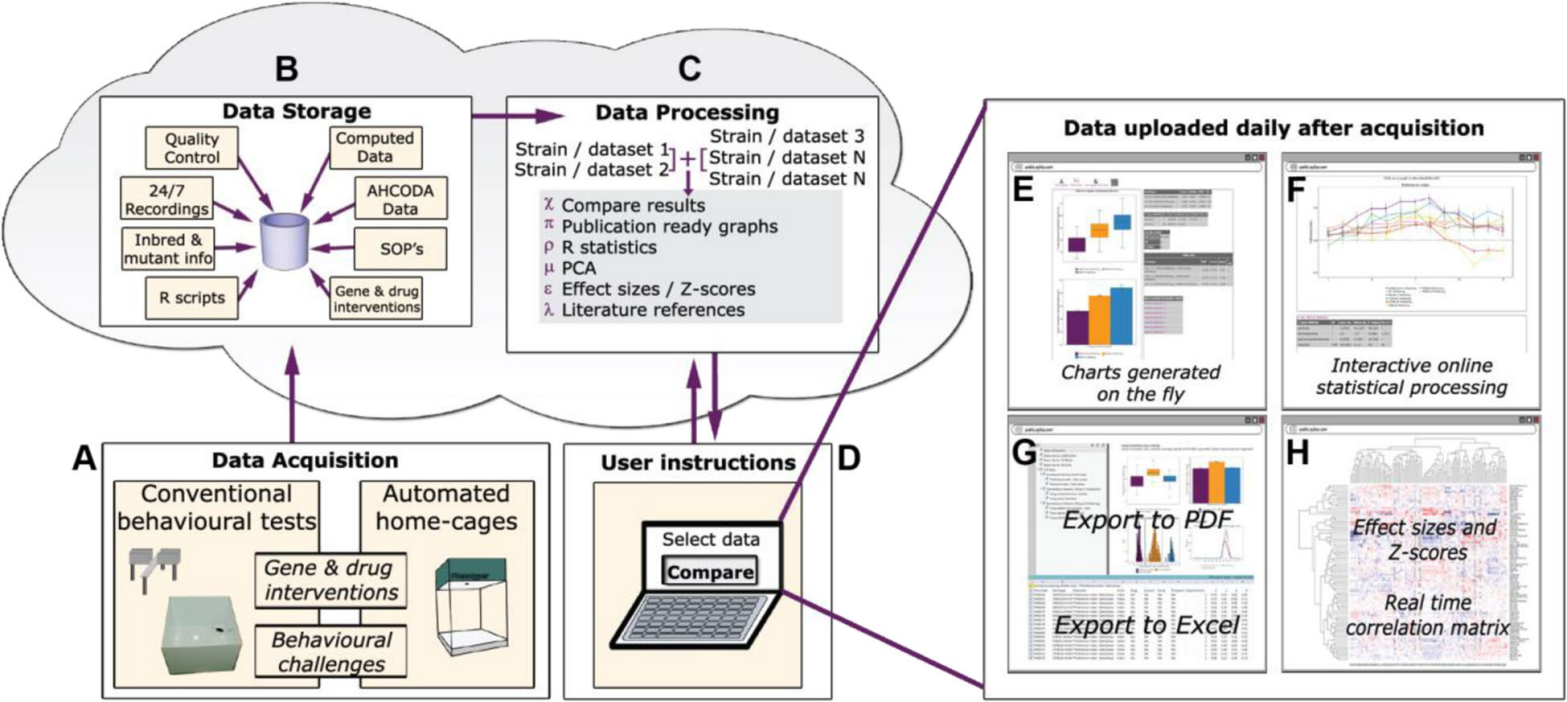
Schematic overview of the workflow underlying the AHCODA-DB repository and website. After data of conventional behavioural tests and automated home-cages is acquired (**a**), the data are transferred to a MySQL database that includes metadata on mice, behavioural tests and analysis parameters (**b**). Data is processed by R-scripts (**c**) selected from user instructions in the AHCODA-DB website interface (**d**). Results of group comparisons are shown in the web browser as publishable ready art and statistics (**e**-**f**) that can be downloaded as a PDF or CSV file (**g**). The heat map function allows large-scale group comparisons (**h**)

The AHCODA-DB repository (MySQL database) contains phenotypic data of mice collected from standard batteries of conventional behavioural tests as well as from automated home-cage experiments (Fig. 1a). Raw data from automated home-cage experiments, executed in any lab running compatible home-cage testing protocols, can be uploaded automatically when the experiment has finished (see the “about AHCODA-DB” page of the website for more detailed information). The raw data from conventional behavioural tests are exported from the tracking software, and imported in the database by the experimenter. Besides raw behavioural data of individual mice, metadata are stored, such as strain/mutation, drug treatment, gender and age, all with unique identifiers (Fig. 1b), as well as a plain text field in which additional non-structured metadata can be stored (e.g. order of testing, details on housing conditions). In addition, the repository contains information on the standard operating procedures (SOPs) of the conducted conventional behavioural tests and protocols used in automated home-cage systems. Common ontologies to describe the behavioural phenotypes, adopted from the Mouse Genome Database (MGD) at the Mouse Genome Informatics (MGI) website (The Jackson Laboratory, Bar Harbor, Maine; www.informatics.jax.org), are used to facilitate data integration with other databases. Each behavioural test is linked to data analysis scripts (R scripts; programmed in R statistical package [11]) that check the quality of uploaded data, exclude data using pre-set criteria for each behavioural test, and precompute frequently requested subsets of the data (e.g. time bins) or specific statistical analyses (e.g. effect-sizes and z-scores).

The AHCODA-DB website (programmed in HTML, PHP and JavaScript) is the front-end user interface of the data repository that allows visualisation and statistical analyses of the raw data contained in the repository. Through the user’s action on the website data is retrieved from the MySQL database by PHP, fed into R-scripts that compute statistical tables and produce graphs (PNG), which are subsequently displayed on the website (by PHP). Since the data of various behavioural tests differ in nature (e.g. continuous versus ordinal data, single time-point versus within-subject repeated measures) requests by the user will lead to the selection of an appropriate R-script from a library of scripts stored in the database. For each dataset, the metadata contained in the repository can be viewed by the user by clicking a dedicated ‘Experimental information page’ link, which generates a webpage with both structured (e.g. gender, age; in tables) and non-structured metadata (e.g. textual description of housing conditions).

On March 9th, 2017, AHCODA-DB contained data of 10 mutant mouse lines, 13 common inbred strains and 30 datasets/publications. These datasets contain 640,246 data points from 2419 parameters and 1563 mice (for details see Tables 1 and 2). The database is constantly updated with new data and the current data content is indicated on the “about AHCODA-DB” page of the website.

**Table 1:** Overview of available experiments in the database with the number of plots

Conventional behavioural experiments		Automated home-cage experiments	
Experiment	# of plots	Experiment	# of plots
Balance Beam	6	Spontaneous behaviour (Activity bouts)	14
Barnes Maze	12	Spontaneous behaviour (DarkLight index)	15
Body weight	4	Spontaneous behaviour (Habituation)	28
Dark/Light Box	12	Spontaneous behaviour (Kinematics)	28
Elevated Plus Maze (5 min)	15	Spontaneous behaviour (Light/dark phase transition pattern)	16
Fear conditioning	26	Spontaneous behaviour (Sheltering)	14
Grip strength meter	4	Appetitive conditioning (pellet task)	1
Morris Water Maze	11	Avoidance learning (shelter task)	8
Nesting	2	Initial discrimination and reversal learning (CognitionWall)	36
Novel Home Cage Induced Hypophagia	1	Mouse characteristics	3
Novel Object Recognition	41	PhenoTyper data per 1-h time bin	2
Open Field	11		
Pre-Pulse Inhibition	10		
Rotorod	7		
T-maze (spontaneous alternation)	4		
Three Chamber Test	13		
Vision Test	1

**Table 2:** Overview of publicly available studies with the number of mice used for the study

Studies publicly available	
Study name/publication	# of mice
129S1/SvImJ reference	62
5xFAD reference	34
A/J reference	52
AppPs1 reference	63
BALB/cJ reference	47
C3H/HeJ reference	29
C57BL/6 J reference	111
CAST/EiJ reference	17
Cognitive flexibility deficits in a mouse model for the absence of full-length dystrophin.	72
DBA/2 J reference	44
Diazepam sedation reference	96
Enhanced alcohol self-administration and reinstatement in a highly impulsive, inattentive recombinant inbred mouse strain.	9
Epileptiform Activity and Cognitive Deficits in SNAP-25 Heterozygous Mice are Normalized by Antiepileptic Drugs.	24
Functional characterization of the PCLO p.Ser4814Ala variant associated with major depressive disorder.	32
FVB/NJ reference	54
Genetic mapping in mice reveals the involvement of Pcdh9 in long-term social and object recognition, and sensorimotor development.	35
GSK189254	208
Hyperactivity, perseveration and increased responding during attentional rule acquisition in the Fragile X mouse model.	54
MK-801 reference	72
MPTP reference	51
Neuregulin-3 in the Mouse Medial Prefrontal Cortex Regulates Impulsive Action.	40
NOD/LtJ reference	47
PCP reference	96
PWK/PhJ reference	16
SOD1 reference	146
Tomosyn-2 is required for normal motor performance in mice and sustains neurotransmission at motor endplates.	38
WSB/EiJ reference	14

## Utility and discussion

### Visualisation and statistics

The AHCODA-DB website is a unique service as it displays high resolution data from behavioural tests where the results, graphs and statistics are generated upon request using R scripts that are selected in response to user instructions on the website (Fig. 1c-d). The major advantage of this approach is that users are able to perform customised analyses on selected data in the repository and visualize the results instantaneously as group means or as individual mouse data, thereby retaining data on variance and potential outliers (Fig. 1e-h). Multiple datasets can be selected for online comparative quantitative assessment, and resulting charts as well as the tables with results of statistical testing of group differences in user-selected behavioural tests are generated on the fly. Depending on the selected data, box plots and bar graphs and respective parametric and non-parametric statistics are presented (Fig. 1e), or in case of longitudinal data, line plots with repeated measures statistics are presented (Fig. 1f). Besides browsing data online, PDF reports can be downloaded in which the charts and results tables of group comparisons in multiple behavioural tests are aggregated, together with the detailed description of the experiment and testing methods (Fig. 1g, upper part). In addition, Excel files can be downloaded that contain the raw data (individual mouse data points) of the selected group comparisons and behavioural tests (Fig. 1g, *lower part*).

The heat map functionality on the AHCODA-DB website (Fig. 1h) enables users to execute more systematic and large-scale comparison of common mouse lines, mutant mouse strains and/or drug effects across the available behavioural parameters. These heat maps visualize effect-sizes, i.e. display the difference between a group of mutant mice and their respective wild type littermates or a drug-treated versus vehicle-treated group, for a user-defined selection of behavioural parameters. Hierarchical clustering of the heat map data allows to systematically compare and group mouse models and drug effects on the one hand, and behavioural parameters obtained in various behavioural tests (conventional and automated) on the other.

### Interpretation

To serve users that are not experts in the field of mouse behaviour, or users that are interested in a precise description of the methods used, detailed information of each behavioural test is available on the website. In addition, for every test parameter used a detailed description has been added for interpretation of the results.

For each published dataset, a summary report of the respective manuscript is available or a link is provided to the publishers website. These reports also contain hyperlinks to key graphs and statistics of the manuscript that substantiate the conclusions.

## Conclusion

The ongoing production of high-content datasets and integration in AHCODA-DB allows –omics scale comparison of behavioural tests, mouse phenotypes and pharmacological responses. By the unbiased publishing of both positive and negative results, AHCODA-DB facilitates scientists in reducing animal usage by avoiding unnecessary repetition of experiments. Furthermore, implementation of standardized quality control and pre-set exclusion criteria contribute to the robustness of the data. The integration of data obtained from different phenotyping platforms, in both common inbred strains as well as mutant lines, with and without drug treatments, increases the scientific value of this open-access repository. Through its easily accessible web interface and various data analysis and mining opportunities, this repository will also increase cost efficiency of publicly funded mouse screening projects and help to reach consensus conclusions on drug responses and mouse phenotypes.

## Availability and requirements

The website is publicly accessible through https://public.sylics.com and can be viewed in every recent version of all commonly used browsers.

## Abbreviations

AHCODA-DB: Automated home-cage observation and data analysis database
CSV: Comma-separated values
HTML: HyperText markup language
MGD: Mouse genome database
MGI: Mouse genome informatics
PDF: Portable document format
PHP: PHP: Hypertext Preprocessor
PNG: Portable network graphics
SOP: Standard operating procedures
